# Electron guiding in macroscopic borosilicate capillaries with large bending angles

**DOI:** 10.1038/s41598-021-87156-4

**Published:** 2021-04-16

**Authors:** Hai-Dang Nguyen, Jan-Philipp Wulfkühler, Jörg Heisig, Martin Tajmar

**Affiliations:** Institute of Aerospace Engineering, Technische Universität Dresden, 01307 Dresden, Germany

**Keywords:** Materials science, Physics

## Abstract

This work presents experiments about the transmission of electrons with an energy of around 15 keV with beam currents up to 20 µA through macroscopic glass capillaries. A systematic study was conducted to experimentally investigate the transmission of electrons through borosilicate glass capillaries with curve angles of 90°, 180°, 270° and 360° for the first time. The focus of the work was to identify the conditions under which the injected electron current is transmitted through the capillary. It was also shown that the transmission process in the macroscopic capillaries can be optically observed by cathodoluminescence—the interaction of electrons with the capillary surface causes locally a blue glow. Different distinctive “glow states” were observed and are found to correlate with different states of electron transmission.

## Introduction

While the standard method to steer the trajectory of charged particles is by means of electrostatic and magnetic optics, insulating capillaries also show the ability to deflect and focus injected charged particle beams. This phenomenon was first observed nearly two decades ago by Stolterfoht et al. when highly charged ${\mathrm{Ne}}^{7+}$-ions with a beam current in the nA-range were found to be guided through a PET nanocapillary foil keeping their initial charge state and energy although the capillary axis was tilted in a manner, that the incoming ions were geometrically unable to pass the capillaries in a straight line^1^. Since then, tests with different ion species, particle energies, capillaries ranging in size from nanocapillaries to macrocapillaries and different geometries (straight, curved, tapered), materials like ceramics, polymers and glass and energies in the range from several hundred eV to MeV have been done. For this, we refer to an extensive review paper by Stolterfoht et al.^2^ and some newer papers^3,4^. The transport of ions through the capillaries has been mostly attributed to charge patches at the inner capillary surfaces created from part of incoming ions. These patches repulse other incoming ions and prevent them from colliding with the walls. Several simulations^5–8^ have aided in understanding this effect which has become known as “ion guiding”.

Similar research has been carried out with electrons that passed through dielectric capillaries ranging from foils with arrays of holes with nanometer sized diameters to single capillaries with lengths of several centimeters and diameters ranging from micrometers to several millimeters.

First investigations of this kind were performed in 2007: At the University of Belgrade Milosavljević et al. irradiated Al_2_O_3_ nanocapillaries with slow electrons with energies of 200 to 350 eV^9–11^. At the Western Michigan University, several experiments with the transmission of slow electrons (≤ 1000 eV) were conducted with PET nanocapillaries and subsequently extended to polycarbonate nanocapillaries and glass macrocapillaries with straight and tapered geometries^12–17^. Additional simulations were performed by the Vienna University of Technology to investigate the underlying mechanisms^18,19^.

Several experiments were carried out at the Moscow State University and at the Belgorod State University. Interestingly, during many experiments the electron energy was higher on the order of 10 keV^20^. These tests began in 2010 with experiments on the transmission of electrons through several macro-sized tubes, polycapillary columns and cones with electron energies between 2 to 10 keV and currents of up to several µA^21^. Subsequent tests included more tests on tubes, tapered capillaries and PET nanocapillaries^22–25^.

The investigations were also extended to curved macrocapillaries. Wang et al.^26^ conducted experiments with a 15° curved SiO_2_ tube (inner diameter of 2.3 mm, total length of 50 mm) and observed the transmission of electrons in the energy range of 1100 to 1500 eV. Measurements with a position sensitive Faraday cup showed a symmetric angular distribution of the transmitted electrons, which was interpreted by Wang that a certain fraction of the electrons is transmitted without collisions through the capillary. However, no direct measurements of the energy of the transmitted particles were conducted. Petukhov et al.^27^ characterized the transmission properties of macroscopic glass and polyurethane tubes (inner diameter 4 mm, bending radius 210 mm) with curve angles of 0°, 45°, 90° and 180° in the energy range of 10 to 20 keV with currents of 5 to 50 µA. The transmission coefficients and the energy spectra were measured with a cylindrical electrostatic analyzer and the X-ray spectra produced by electrons exiting the tube were also investigated. The current transmission factor did not so much depend on the initial energy of the electrons but dropped from approximately 80% at 0° to below 50% at 90° bending angle°. However, the electrons lost kinetic energy during the transmission process: For higher bending angles the maxima of the transmission spectrum energies dropped to lower energies, the spectrum broadened with a significant increase of low energy electrons.

The electron transport process is still not fully understood but believed to be based on a combination of small angle elastic and inelastic scattering of the electrons with atoms in the bulk material of the capillary walls as well as similar to ion guiding formation of charge patches in deeper layers of the capillary surface. This is based on the observation of elastic as well as inelastic transmitted electrons and a measured time delay between the injection of electrons and the transmission at the outlet of capillaries^4,14,28^. This means that the transport of electrons through dielectric capillaries significantly differs from the ion guiding mechanism. Nevertheless, the term “capillary guiding” is understood to describe the transmission of particles along the capillary axis, whether they are deflected electrostatically by charge patches or by scattering on atoms of the bulk material. Only secondary electrons are specifically excluded from this definition^2^. Thus the transmission of electrons through capillaries will be referred to as “electron guiding”.

In this work we describe the transmission of electrons through curved macrocapillaries with bending angles up to 360° and currents of around 20 µA. By doing so, we significantly expanded the range in which the successful transmission of electrons has been described in the open literature. Furthermore, as we will discuss in this work, large transparent capillaries enable a new approach for the investigation of the guiding effect for electrons because the transmission process is accompanied by the local emission of light in the visual range from cathodoluminescence, indicating regions in the capillary where electrons hit the wall. This work is mainly focused on observing these two aspects without investigating the energy spectrum of the transmitted particles. Therefore, we cannot make definite statements about the underlying transmission mechanism. Energy measurements are planned for future tests.

This paper is structured as follows: The next two sections describe the experimental setup and the capillary samples. Then, the experimental results are presented and discussed in a separate section. We conclude with a summary and outlook.

## Experimental setup

For the electron guiding experiments the glass capillaries were tested one after another in a cylindrical vacuum chamber (Ø0.9 m × L1 m) equipped with a Pfeiffer HiPace 2300 turbomolecular pump capable of providing a base pressure of 1E-6 mbar. A viewport in the chamber allowed the visual observation of the experiment.

The experimental setup consists of the capillary itself, which is mounted to a holder. An electron source is positioned in front of the capillary inlet. A target electrode is placed in a certain distance from the exit of the capillary to collect the transmitted electron current. An overview of the setup is shown in.

A custom made hot filament electron source consisting of a v-shaped tungsten filament enclosed by a Wehnelt cylinder and a grounded acceleration electrode was used for the experiments. The filament is capable of emitting several hundreds of microamperes. The acceleration voltage is up to 16 kV.

The capillaries were mounted to an insulating holder at the inlet region. An aperture system is placed in front of the inlet. It consists of two electrodes which are electrically insulated from each other by a 3 mm thick insulating spacer. The first electrode consists of a graphite sheet and has as an aperture diameter of 4 mm (and is therefore smaller than the capillary channel). It faces towards the electron source and collects any currents emitted by the electron source which do not enter the capillary. This way the maximum electron beam divergence becomes 5 degrees in a distance of 50 mm to the electron source. The second electrode is directly attached to the capillary inlet with an aperture diameter of 6 mm and collects the current that enters the capillary but is not transmitted. It therefore acts as “back-flow” collector. An offset voltage was created between the Wehnelt cylinder and the filament by inserting a pre-resistor in the range of 100 kOhm to 1 MOhm. This provided further control over the beam shape and the current density. The emission current from the electron source ${I}_{e}$ is either collected by the aperture, referred to as aperture current ${I}_{ap}$ or injected into the capillary denoted as injection current ${I}_{inj}$. The fraction of the injection current that is actually transmitted and detected by the target is referred to as target current ${I}_{t}$. The remaining back-flow current ${I}_{bf}$ is collected by the back-flow electrode. The electron current balance can therefore be stated as follows:

1 $${I}_{e}={I}_{ap}+{I}_{inj}={I}_{ap}+{I}_{bf}+{I}_{t}$$

**Figure 1:**
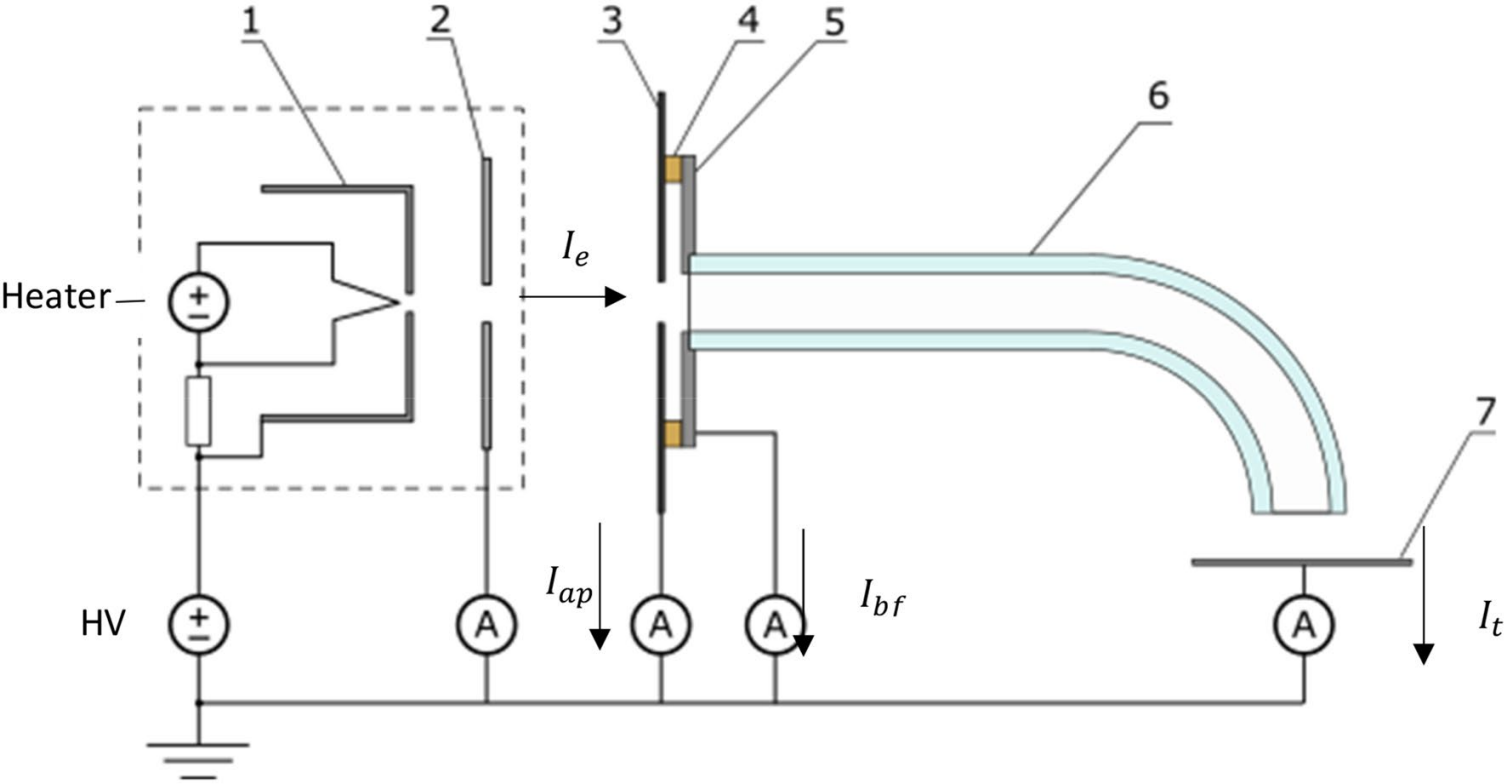
Layout of experiments with electron source (in dashed box) and capillary setup. 1. Wehnelt cylinder, 2. Extractor electrode, 3. Aperture, 4. Insulating spacer, 5. Back-flow electrode, 6. Capillary sample, 7. Target electrode.

The transmitted current ${I}_{t}$ is collected by an electrode with a graphite surface approx. 10 mm downstream of the capillary. In general such a solution is not advisable and a Faraday cup is preferred as the measurements are influenced by secondary electron emission and backscattering. By measuring the current flow from all of the electrodes as shown in Fig. 1 it was possible to create a current balance and estimate the error of the target current caused by secondary electron emission and backscattering to around 10%. A factor $\eta$ representing the transmission efficiency for the capillary was defined as follows:

2 $$\eta =\frac{{I}_{t}}{{I}_{inj}}=\frac{{I}_{t}}{{I}_{bf}+{I}_{t}}$$

The currents were measured with a LabJack T7 pro based data acquisition system with a resolution of 0.1 µA and 5 Hz.

Preparatory to installing the capillaries into the setup, efforts were made to remove any residual charges on the inner and outer surface of the capillaries that may have built up during handling and storage by cleaning the surface with isopropanol.

## Capillary samples

For the experiments the focus lied on the transmission of electrons through macroscopic capillaries with different curve angles. To limit the amount of parameters, capillaries with different bending angles were manufactured that all shared the same inner diameter of approximately 6 mm, a wall thickness of 2 mm, a curve radius of 140 mm and a straight inlet region with a length of 150 mm. The inner diameter of the capillaries together with the straight inlet region ensured a large aspect ratio (total length of the capillary divided by its inner diameter) for all capillaries. Samples with the following curve angles were custom manufactured: 90°, 180°, 270°, 360° and their geometric properties are summarized in Table 1. Borosilicate glass was chosen due to its robustness and affordability.

**Table 1:** Overview of borosilicate glass capillary samples. They all have an inner diameter of 6 mm, an outer diameter of 10 mm and a curve radius of 140 mm.

Curve Angle	Inlet Length	Total Length	Aspect ratio
[°]	[mm]	[mm]	[−]
90	150	370	61.7
180	150	590	98.3
270	150	810	135.0
360	150	1030	171.7

## Experimental results

### Electron transmission

Four different capillaries were tested in sequence from the smallest curving angle (90°) to the largest (360°). Due to time constraints every capillary was tested only once.

**Figure 2:**
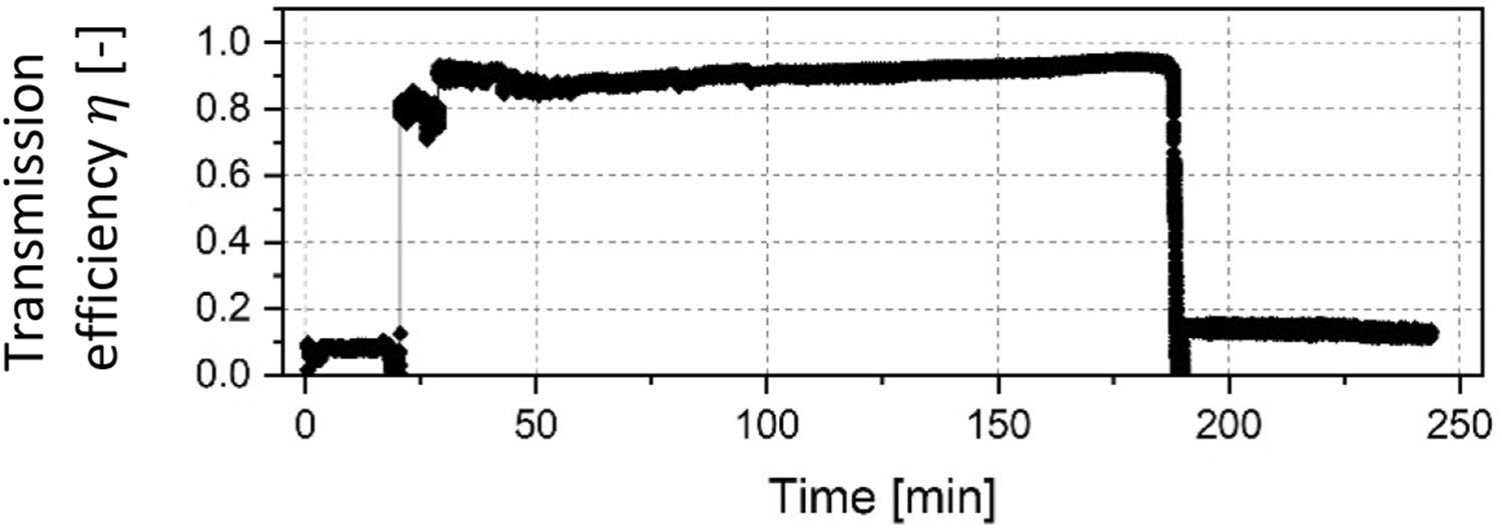
Transmission efficiency through a 90° capillary with a primary particle energy of 14.8 keV and an injection current of 15 µA.

The first test was carried out with the cleaned 90° capillary in which an electron beam with an energy of 14.8 keV was injected. To create a more narrow beam, an off-set voltage of ${U}_{W}$ = − 45 V was applied to the Wehnelt cylinder of the electron gun. Figure 2 depicts the development of the transmitted current over time.

The total testing duration was 4 h to investigate long term behaviour of the guiding performance. It can be seen, that the transmitted current was very low at the beginning when about 2 µA reached the target, which is assumed to be the result of small angle scattering processes and secondary electron emission inside the capillary. At the 20 min mark, the target current fell close to zero for a few seconds, then abruptly rose to about 94% of the maximum injected current of 15 µA and afterwards the electron transmission was stable for the next 160 min, as the data in Fig. 2 shows. After around 2.5 h the transmission broke down suddenly and afterwards only 15% of the injected current reached the target.

**Figure 3:**
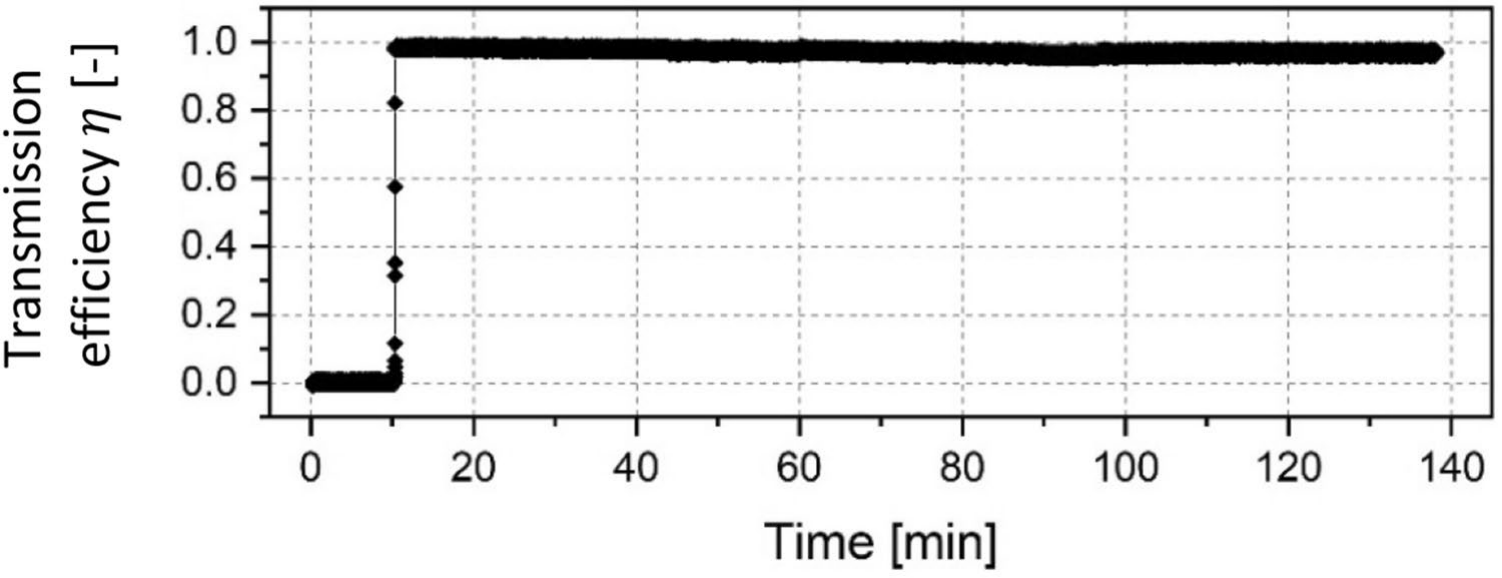
Normalized current transmitted through a 180° glass capillary with a primary particle energy of 15.5 keV. After around 140 min the on–off tests were conducted (see Fig. 4).

**Figure 4:**
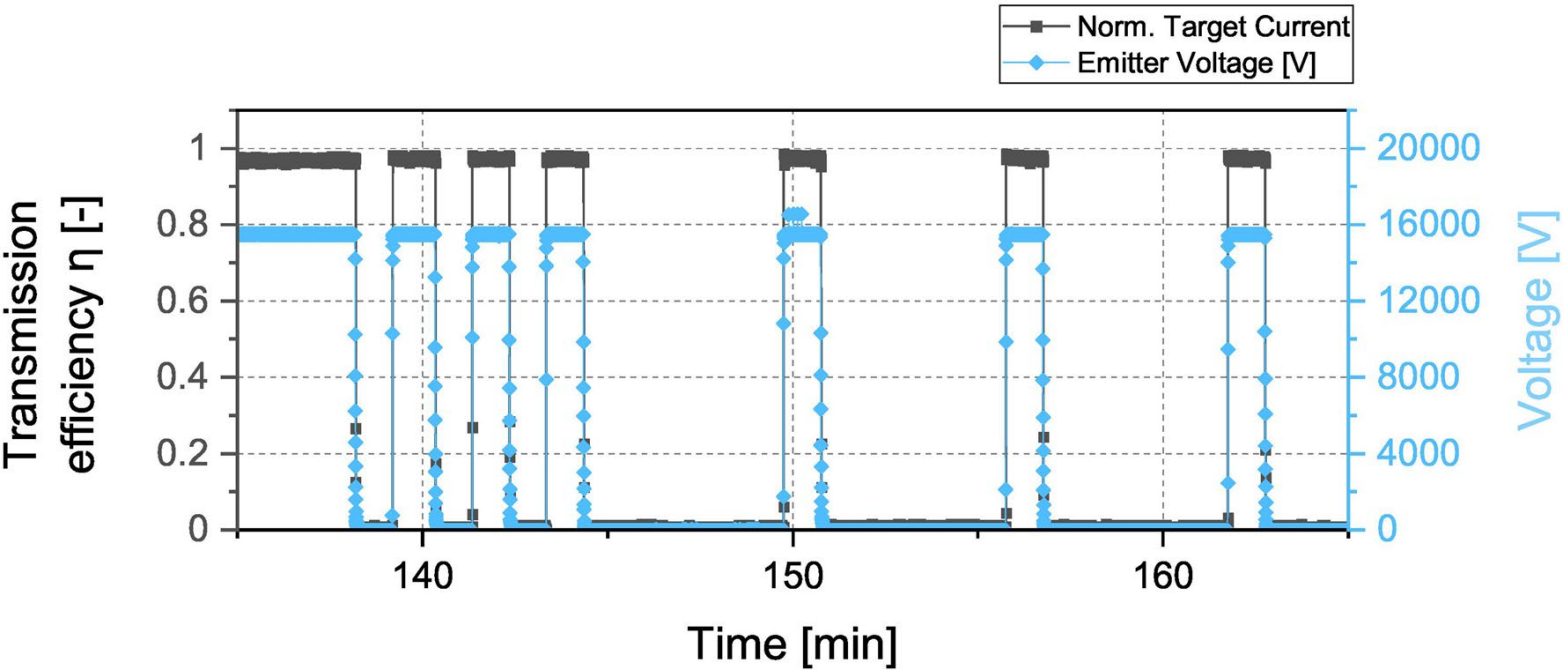
On–off tests with a 180° capillary. The black graph depicts the transmission current whereas the blue graph represents the applied acceleration voltage of the electron gun which indicates the turn on and turn off of the electron beam.

When testing the 180° capillary, we faced some issues concerning the beam injection as unfortunately the capillary inlet and the electron beam were not properly aligned. Hence an adjustment was made where the electron beam was broadened by reducing the offset voltage on the Wehnelt cylinder to ${U}_{W}$ = -14 V which created an injection current of about 3.3 µA. The primary energy of the electron beam was 15.5 keV and the normalized transmitted current is shown in Fig. 3. Contrary to our expectations, the 180° capillary showed a behaviour similar to that of the 90° capillary despite its inlet not being properly aligned with the electron beam. After a time delay of about 10 min, the target current abruptly increased to a value of around 98%, which was remarkably stable for the total testing time of 120 min. Preliminary on–off tests were carried out in which the electron beam was alternately turned off and on for 60 s each. This was repeated 3 times and afterwards the off-interval was extended to 300 s, which was also repeated 3 times as depicted in Fig. 4. It can be seen, that the transmission efficiency rose instantly to the same value for every time the beam was turned on while no time delay was observed.

**Figure 5:**
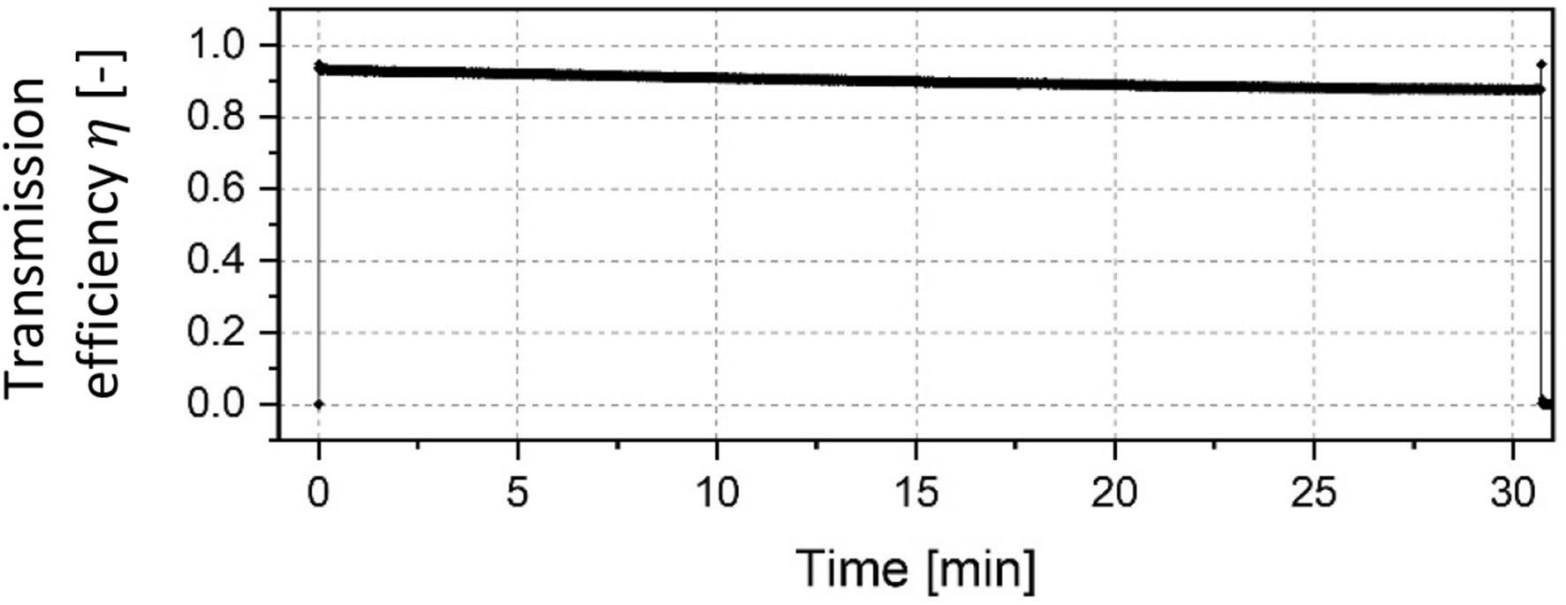
Normalized current transmitted through a 270° glass capillary with a primary particle energy of 14.5 keV.

For the test including the 270° capillary the electron beam was narrowed again by applying an off-set voltage of ${U}_{W}$ = − 60 V, to create similar conditions as in the 90° capillary test. In this configuration, we injected around 10 µA with an energy of 14.5 keV into the sample for about 2.5 h. During this whole period, the target current remained zero and no electron transport through the capillary could be observed. To examine the dependence of the beam shape on the guiding process, the electron beam then was turned off and the offset voltage was reduced to ${U}_{W}$ = -14 V. After the electron beam was turned on again, instant transmission occurred through the 270° capillary with an efficiency of around 93% which slowly decreased to 87% after 30 min as displayed in Fig. 5.

On–off tests were carried out similar to those for the 180° capillary with similar results which supports the model of charge patch assisted guiding.

**Figure 6:**
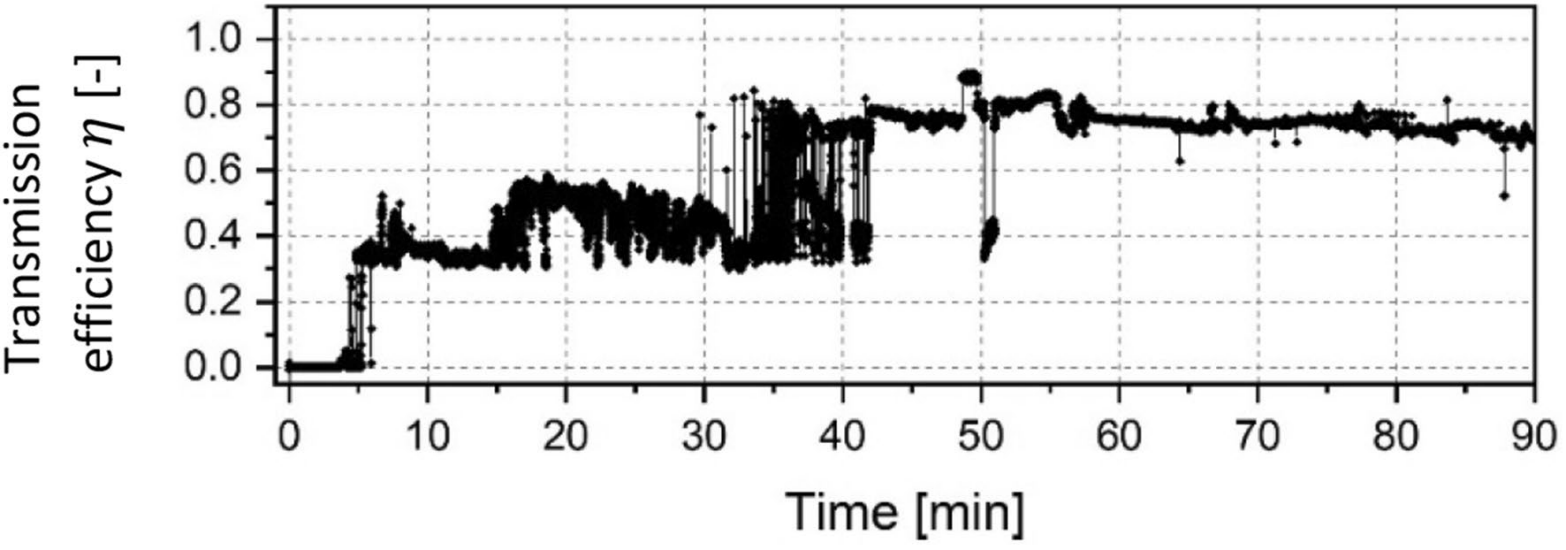
Normalized current transmitted through a 360° glass capillary with a primary particle energy of 14 keV.

In Fig. 6 the data of successful electron transmission through the helix capillary with a 360° curve angle is displayed. Due to the results of the previous test, a similar beam configuration was applied here. Before injecting the current, efforts were made to free it of any residual electric charges on the inner and outer surface. Comparable to the 90° and 180° tests, there was a time delay before significant transmission started. Interestingly, the “quiet period” was even shorter than that of the 90° and 180° capillary. But unlike in the previous tests, in which the transmitted current abruptly increased to a value of more than 90%, the guiding behaviour for the 360° capillary was clearly different. First, the efficiency of the initial electron transport was at about 33% for around 10 min. After that the guiding process was accompanied by an excessive noise. After passing the 44-min mark, the transmission process reached an equilibrium and the transmitted current was somewhat stable at around 74% until the test was stopped after 90 min (with one major exception at 50 min). The results of the on–off tests for this capillary were similar to the previous ones and showed that the transmission efficiency did not change much even after turning the electron beam off for a few minutes.

### Observation of cathodoluminescence during transmission

**Figure 7:**
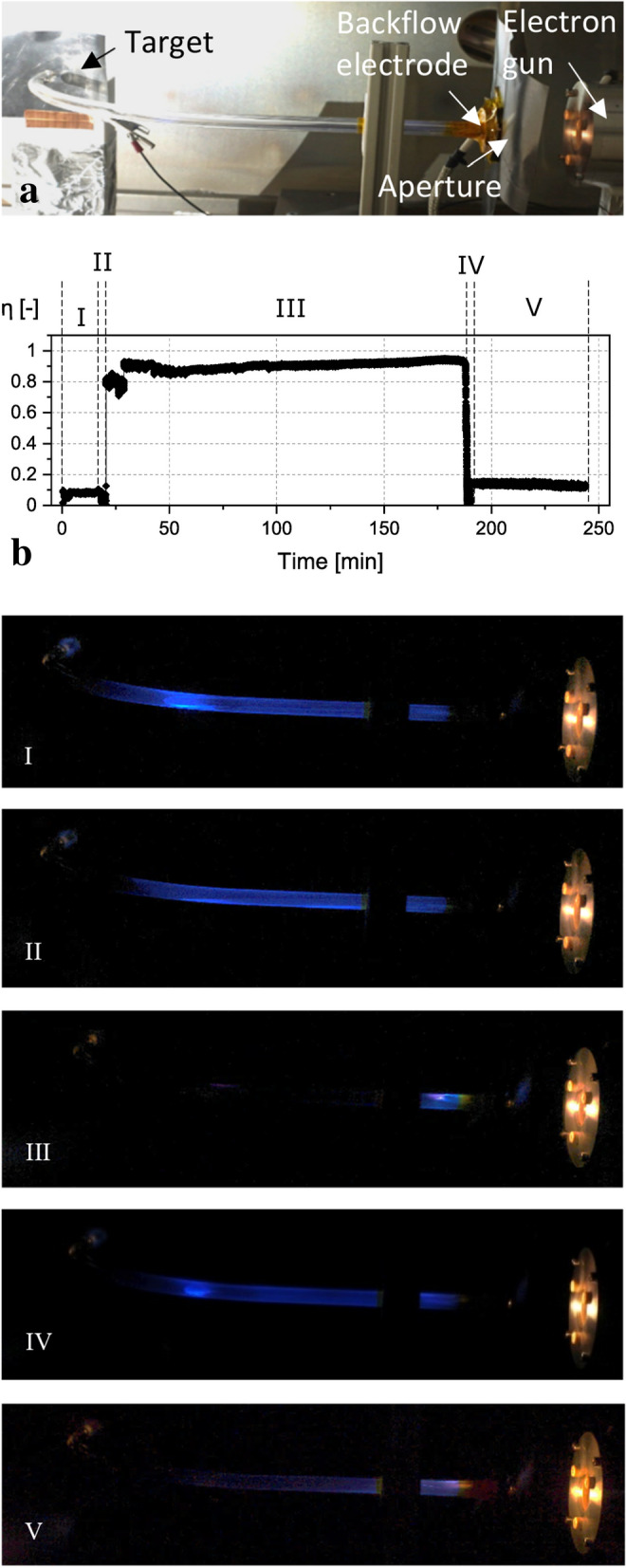
Photos of different glow states during the guiding test through a 90° capillary. (**a**) General Setup (**b**) Different guiding stages for the 90° Capillary, I: Poor transmission, II: 3 min before guiding started, III: During guiding process, IV: Guiding was interrupted, V: After guiding was interrupted.

A blueish glow of the capillaries could be observed in the course of all tests. This is most likely attributed to the cathodoluminescence of glass where impacting electrons lead to the creation of electron–hole pairs in the material which subsequently recombine and emit photons in the visible spectrum^29^. Throughout the tests, we were able to observe different glow states which appear to correlate with the electron transport. The 90° capillary and the 360° capillary were chosen for a detailed investigation of this phenomenon. Although the bright patches at the inlet and curved parts of the capillaries could be observed with the naked eye, the photos were taken with high exposure times, which made the glow pattern in the capillaries better visible. An f-number of F/3.5, a shutter speed of 30 s and an ISO value of 100 were used. As far as we know, there are no studies concerning an optical observation of the electron guiding effect yet. In Fig. 7 different glow states of the earlier mentioned 90° capillary test are displayed for certain moments after the irradiation was started.

Figure 7a shows the general setup for the 90° capillary as explained earlier including the target, the backflow electrode, the aperture and the electron gun. A narrow beam was injected into the capillary with a primary particle energy of 14.8 keV. In Fig. 7b the transmission process in subdivided into different guiding stages. The initial glow state of the capillary during the first 10 min is depicted in Fig. 7-I. It can be seen, that the whole straight section and a part of the curved section is glowing. A clear and more intense blue spot is visible at the start of the curvature, as this is the region where most of the electrons are likely to impact the capillary wall. This spot disappeared during the next minutes, as depicted in Fig. 7-II. Although two smaller spots are visible, the straight entrance section and the beginning of the curvature glow mostly uniformly. This state occurred around 3 min before more efficient electron transmission started. The evenly glow might be attributed to the charge up of the capillary, which lead to a more uniform distribution of impacting electrons on the capillary wall. Figure 7-III displays the exciting moment when the transmission efficiency instantly rose and electron guiding occurred. The glow inside the capillary surprisingly reduced almost instantly to an intense glow only at the start of the capillary entrance with an additional very weak spot in the region where the curvature starts. This state remained mostly unchanged until the electron transport was interrupted. Right afterwards the glow state was very similar to the one at the beginning of the test with an intense blue spot at the curvature (Fig. 7-IV). This appearance slowly reduced into a weaker uniform purple glow in the entrance section without any noticeable spots, as displayed in Fig. 7-V. This might correspond to the mentioned blocking state, in which an overcharged capillary hinders further electron transport through the capillary. Afterwards, in this experimental configuration the electron transmission remained low at about 15%. Additional tests in which the primary energy of the injected electron beam was varied, showed a correlation between particle energy and intensity of the emitted light, while the colour remained seemingly unchanged. Hence, additional work is required to examine the reason for the change in colour after the guiding process collapsed.

**Figure 8:**
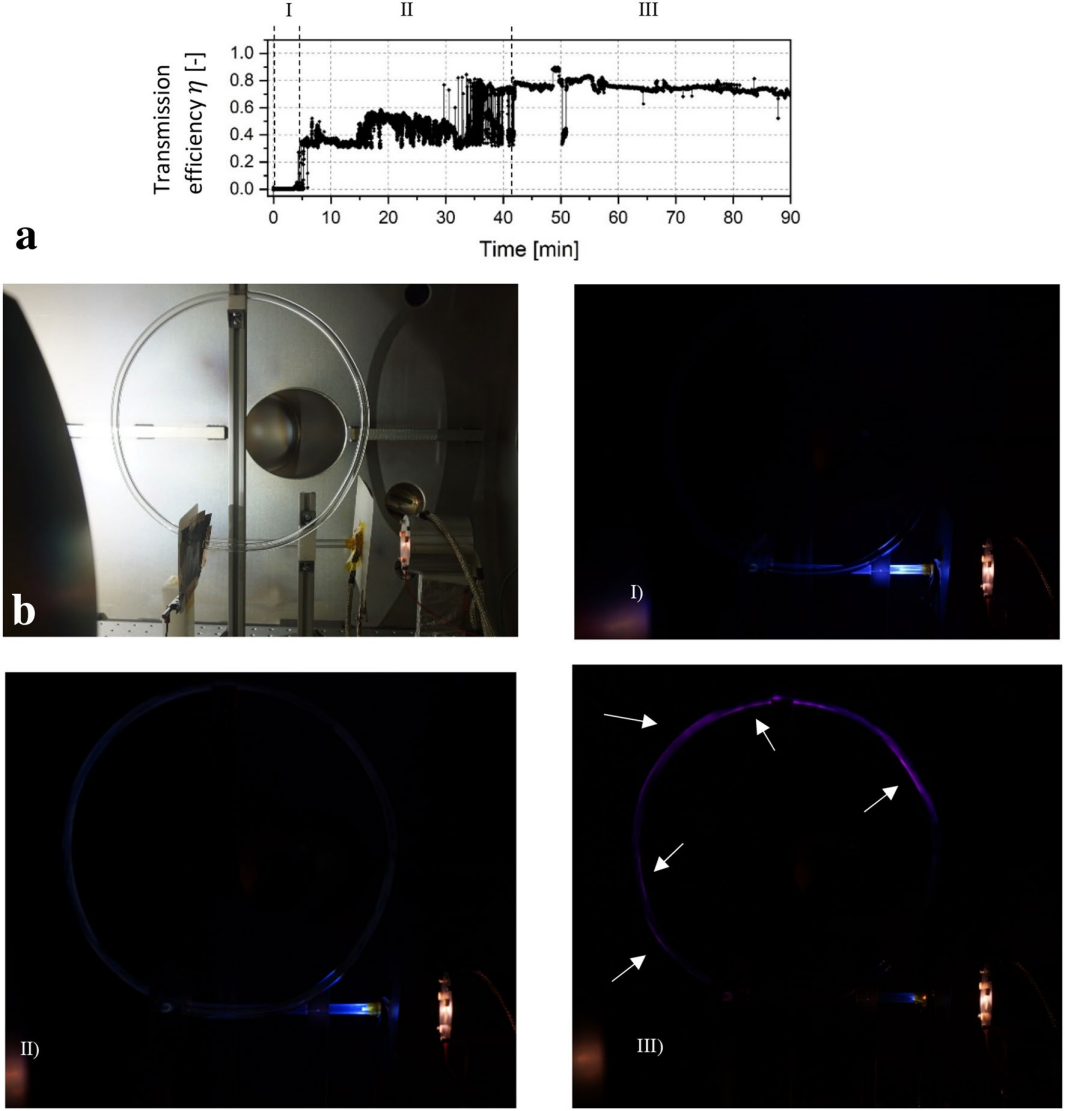
360° capillary and its glow states: (**a**) Data plot with different guiding stages, (**b**) General setup, (I) Initial glow state before guiding, (II) Intermediate phase with dynamic electron transmission, (III) During Equilibrium. The arrows point on visible glow spots formed inside the capillary.

The 360° capillary was mounted in a way that every section could be observed, as depicted in Fig. 8. Before guiding was achieved, a blue glow at the entrance section and the start of the curvature was visible (see Fig. 8-I). Unlike in the 90° capillary, there was no intense spot in the bent part of the helix. Instead, the highest intensity was visible at the inlet region of the capillary, which is most likely attributed to the fact that a broadened beam was injected as explained earlier. Figure 8-II shows the change of the visible light formation, when electron transport was achieved at the 5-min mark. While the intensity in the straight section was strongly reduced, the whole capillary glowed very weakly and evenly, which may be due to low energetic particle wall interactions. While the electron transport was somewhat stable at the beginning, it was accompanied by a growing noise during the next minutes. The strong noise in the current measurements indicated a dynamic evolution of additional negative charge patches before reaching equilibrium. In this stage the entrance region still glowed blue and multiple purple spots slowly formed visibly at different regions in the capillary which could be an optical indicator for the charge distribution. These spots were visible in the first 270 degrees of the capillary while there was no noticeable glow in the last quarter section. The blue glow at the outlet is most likely the reflection of the light in the inlet. When transmission reached equilibrium after around 42 min, the spot formation remained mostly unchanged. Figure 8-III shows a picture of the capillary during equilibrium.

## Discussion

In the discussion of the experimental observations we want to focus on the five following aspects:

1. The successful transmission of electrons through all capillary configurations.
2. The observed time delay before the start of the transmission
3. Blocking of capillary after long-time transmission
4. Stable transmission in on–off tests
5. Visual observability of guiding process due to cathodoluminescence.

Electron transmission through all four capillary configurations was achieved in all tests with a particle energy of approximately 15 keV. This value was determined from preliminary tests. Other parameters varied: For example the currents that were injected into the capillaries varied from 3.3 µA to 20 µA and the transmission efficiency was found to be between 73% to nearly 100%. Due to a lack of a device to measure the particle energy of the transmitted electrons, it is not possible to clearly define if the particles were transported through the dielectric tubes primarily be elastic scattering, inelastic scattering or the guiding from assembled negative charge patches. Future tests will have to show if there is a minimum kinetic energy for the transmission process.

The irradiation of the capillaries showed a significant time delay before efficient guiding was achieved. One exception was the 270° capillary which was irradiated with two different beam configurations before the current was transmitted. Therefore, no exact charge up phase could be determined for this sample. In studies performed with nano- and microsized capillaries, the time delay before high transmission was a clear indicator for the build-up of negative charges on the inner capillary surface assisting the electron guiding process. This might also be the case in our experiment. The build-up of a charge distribution could lead to the deflection of further incoming particles towards the capillary exit. A long-term test with the 90° capillary showed a stable electron transport over the course of 2.5 h. An interruption of the electron transport lead to a sudden breakdown of the transmitted current from over 90% to only 15% (see Fig. 2). We considered possible blocking effects that might have disturbed the guiding process due to overcharging as well as sudden discharges of a negative patch within the borosilicate glass capillary as observed in 2010^17^. The scenario of electron transmission after a quiet period has been observed in several studies^14,28^, where electrons were guided through PET nanocapillaries. However, the current densities were much smaller compared with our experiments. Furthermore, Petukhov et al.^27^ mentioned no time delay for the transmission of currents through capillaries with 90° and 180°. The filament electron gun in our experiment is a source of uncertainty. A variable focus was achieved by means of the negative voltage offset created by a pre-resistor of the Wehnelt cylinder. The aperture electrode in front of the capillary inlet provides an additional collimation. But it is unclear, if the local beam current density and beam alignment were similar in all experiments and stable with time. The focus of the electron source could only be significantly changed by changing the pre-resistor, which prevented a dynamic adaption of the focus without switching the source on and off in between.

The current transmitted through the 180° sample showed a more stable behaviour and the quiet period was shorter than for the 90° capillary. It has to be noted, that in this test a much lower current and also a lower current density was injected into the capillary due to misalignment in the setup. These factors appear to have a strong influence on the guiding performance, as the following 270° test showed. Here a focused beam could not be transmitted, whereas an injection with lower current density lead to guiding. This shows that the potential charge distribution inside macroscopic capillaries also depends on a number of beam parameters e.g. the current density, the angle of injection and the beam divergence which affect the charge distribution on the inner capillary walls.

While a quiet period followed by an instant transmission phase was observed in the 90° and 180° samples, the data of the 360° helix capillary test show an intermediate phase in which the transmission was accompanied by strong noise. This is most likely the phase in which the transmission varies as a result of the dynamic evolution of additional negative charge patches, possibly accompanied with sudden discharges as reported by Dassanayake^17^. During the equilibrium the transmission efficiency was at around 74%, which is much lower compared to the previous samples. This might be attributed to the greater capillary length and higher aspect ratio as well as the larger bending angle but has to be examined in further studies.

An overview of parameters used in the presented tests are given in Table 2. A counterintuitive but notable factor is that the duration of the quiet period seems to scale disproportionately with the curve angle. In case of the 360°, the time delay before a significant transmission occurred was only 5 min, while for the 90° capillary it was four times the duration. A possible reason for the longer quiet phase inside the shorter capillary might be the usage of a narrow beam whereas a broader beam was injected into the helical capillary. However, a lot of additional work is required to investigate the influence of varying parameters on the guiding performance and to reduce sources of uncertainty to provide similar conditions for each test.

**Table 2:** Overview of the testing parameters used in the presented electron guiding experiments.

Curve angle	Injected current	Particle energy	Quiet period	Time until equilibrium	Max. transported current
90°	15.0 µA	14.8 keV	20.5 min	20.5 min	94.2%
180°	3.3 µA	15.5 keV	10.3 min	10.3 min	97.8%
270°	12.0 µA	14.5 keV	–	–	93.7%
360°	20.5 µA	14.0 keV	5.2 min	41.5 min	74.1%

Preliminary on–off tests to investigate the durability of the capillary state were carried out. An example of the corresponding data is displayed in Fig. 4 for the 180° capillary. No influence on the guiding performance was observed which indicates that the equilibrium state inside the capillary remained stable to some extent. This was not surprising as experimental and numerical investigations on ion guiding have shown, that discharge times in highly insulating materials are very high and can even reach days^5^. A study on the time dependence of electron transmission through a single glass capillary performed in 2010 reported a decay constant of 8 min^17^. However this experiment was conducted with incident energies of up to 800 eV and in geometrically a smaller sample than in our experiment. Thus we assume that the relaxation time in our case is much larger due to larger geometries and higher incident energies, which in turn lead to larger drift distances and stronger penetration of the particles into the bulk material. That said this subject definitely has to be looked more into in future investigations.

As already mentioned, due to time constraints every sample was only tested once. A study on electron transmission of 10 keV electrons through a tapered glass capillary was conducted by Vokhmyanina et al. in 2018 where transmission was achieved, but the repeatability of the results were not stable^30^. In 2013 an experiment was performed by Dassanayake in which the irradiation of a single glass capillary was repeated after a discharge time of 6 days with differing results^14^. Therefore multiple tests on the same sample with reduced parameters are planned for future experiments to investigate the repeatability and look for further potentially influencing aspects such as possible damage of the capillary material caused by irradiation or the dependence of the guiding performance on temperature and ambient pressure.

The cathodoluminescence effect and its correlation with the transmission stage is in our opinion a very interesting and valuable observation in the research of electron guiding through glass capillaries. The intensity of the glow might give information about the energy of the incident particles. . Also, since the electrons need to hit the wall with sufficient energy, the glow patterns give new information about the transport mechanisms inside the capillary. The following possible explanations might be considered:

- During transmission, the glowing spots are regions where electrons penetrate inside the bulk whereas the particles are deflected above the surface in the dark areas.
- Electrons are also scattered in the dark regions but the incident particles do not transfer enough energy to enable light emission.
- Non-glowing parts of the capillary might indicate regions where the incident electrons are too slow to enable light emission.

The correlation between the glow states and the guiding phases should definitely be investigated more in further research.

## Summary and outlook

Transmission of electrons through macroscopic glass capillaries with curve angles of 90°, 180°, 270° and 360° were tested with a high energy electron beam. Overall stable electron transport of around 74% was achieved in the 360° capillary whereas a transmission efficiency of more than 90% with total currents up to 20 µA was observed in the remaining samples. We also observed a clear time dependence of the transmission similar to the temporal behaviour reported in previous studies with lower energies and smaller capillaries. Due to the overall differences in capillary size and the parameters of the incident particles, it is difficult to apply the results of the studies performed with smaller capillaries one by one. Nevertheless, the observed time delay is a strong indicator that a charge deposition inside the capillary is required to create a field distribution which leads to the deflection of further incoming particles towards the capillary exit. The variation of the beam properties also revealed its influence on the guiding efficiency as the transmission of beams with lower current density was more successful.

A blueish glow of the samples was visible in all tests, which is attributed to the cathodoluminescence of borosilicate glass and is most likely a strong indicator for the interactions of the electrons with the material. The glow was visible in different regions of the capillary samples and the formation transformed depending on the guiding stage. The 360° helix clearly showed different spots, which appear to correlate with the charge distribution and the guiding state of the capillary.

Additional work is required to further examine the electron transport process through bent capillaries. To understand the guiding mechanism, investigation of the energy distribution of the transmitted beam is crucial and will be implemented in future tests. Also additional studies are necessary to investigate the influence of different parameters on the guiding performance, such as primary energy, current density, beam shape, capillary size and many more.

The cathodoluminescence of the capillary samples is an evident indicator for the current guiding stage. It is expected to be a part of future studies concerning the electron guiding through macroscopic glass capillaries to reveal more information about the underlying mechanism of the guiding process.
